# Exercise Training-Related Changes in Cortical Gray Matter Diffusivity and Cognitive Function in Mild Cognitive Impairment and Healthy Older Adults

**DOI:** 10.3389/fnagi.2021.645258

**Published:** 2021-04-08

**Authors:** Daniel D. Callow, Junyeon Won, Gabriel S. Pena, Leslie S. Jordan, Naomi A. Arnold-Nedimala, Yash Kommula, Kristy A. Nielson, J. Carson Smith

**Affiliations:** ^1^Department of Kinesiology, University of Maryland, College Park, MD, United States; ^2^Program in Neuroscience and Cognitive Science, University of Maryland, College Park, MD, United States; ^3^Department of Psychology, Marquette University, Milwaukee, WI, United States; ^4^Department of Neurology, Medical College of Wisconsin, Milwaukee, WI, United States

**Keywords:** diffusion imaging, MCI, physical activity, exercise training, verbal fluency, episodic memory

## Abstract

Individuals with Mild Cognitive Impairment (MCI) are at an elevated risk of dementia and exhibit deficits in cognition and cortical gray matter (GM) volume, thickness, and microstructure. Meanwhile, exercise training appears to preserve brain function and macrostructure may help delay or prevent the onset of dementia in individuals with MCI. Yet, our understanding of the neurophysiological effects of exercise training in individuals with MCI remains limited. Recent work suggests that the measures of gray matter microstructure using diffusion imaging may be sensitive to early cognitive and neurophysiological changes in the aging brain. Therefore, this study is aimed to determine the effects of exercise training in cognition and cortical gray matter microstructure in individuals with MCI vs. cognitively healthy older adults. Fifteen MCI participants and 17 cognitively intact controls (HC) volunteered for a 12-week supervised walking intervention. Following the intervention, MCI and HC saw improvements in cardiorespiratory fitness, performance on Trial 1 of the Rey Auditory Verbal Learning Test (RAVLT), a measure of verbal memory, and the Controlled Oral Word Association Test (COWAT), a measure of verbal fluency. After controlling for age, a voxel-wise analysis of cortical gray matter diffusivity showed individuals with MCI exhibited greater increases in mean diffusivity (MD) in the left insular cortex than HC. This increase in MD was positively associated with improvements in COWAT performance. Additionally, after controlling for age, the voxel-wise analysis indicated a main effect of Time with both groups experiencing an increase in left insular and left and right cerebellar MD. Increases in left insular diffusivity were similarly found to be positively associated with improvements in COWAT performance in both groups, while increases in cerebellar MD were related to gains in episodic memory performance. These findings suggest that exercise training may be related to improvements in neural circuits that govern verbal fluency performance in older adults through the microstructural remodeling of cortical gray matter. Furthermore, changes in left insular cortex microstructure may be particularly relevant to improvements in verbal fluency among individuals diagnosed with MCI.

## Introduction

Mild Cognitive Impairment (MCI) prevalences in adults above age of 65 years is between 10% and 20% (Langa and Levine, 2014). MCI is a transient state between normal aging and dementia, which generally progresses to dementia at an annual rate between 5% and 20% (Langa and Levine, 2014; Jongsiriyanyong and Limpawattana, 2018). Individuals with MCI experience measurable deficits in functional and cognitive domains such as language, attention, reasoning, executive function, and memory performance (Saunders and Summers, 2010; Teng et al., 2013; Lindbergh et al., 2016; Ding et al., 2019). While the cause of MCI remains uncertain, growing evidence suggests that MCI is associated with volumetric loss, vascular pathology, neuroinflammation, and synaptic dysfunction, particularly in the temporal, prefrontal, and insular cortex (Fan et al., 2008; Scheff et al., 2011; Popa-Wagner et al., 2015). Despite this, some people with MCI remain cognitively stable and can even experience improvements in cognitive performance (Kaduszkiewicz et al., 2014). Thus, while there are currently no known treatments for dementia, MCI presents a potential opportunity to implement non-pharmacological interventions that may slow or prevent neurological deterioration and functional decline.

Identifying effective non-pharmacological interventions require the use of measures sensitive to underlying neurophysiological changes that precede gross structural and functional decline. To this end, volumetric measurements are sensitive to changes in the size of cortical and subcortical gray matter and are used extensively to track cognitive decline in aging and dementia. Yet, these macrostructural measures are often not sensitive to early neurophysiological changes in tissue microstructure that are thought to precede volumetric tissue changes. Meanwhile, advancements in diffusion-weighted imaging, now allow researchers to ask questions regarding the composition and microarchitecture of underlying brain tissue (Le Bihan, 2003, 2014; Hansen et al., 2013; Weston et al., 2015; Assaf, 2019). Diffusion imaging probes at microstructural integrity by quantifying the diffusion of water molecules within a voxel, which is used to infer the underlying tissue’s functional and structural properties (Le Bihan, 2003, 2014; Walhovd et al., 2014). Although diffusion imaging has traditionally been used to examine white matter tract structure and integrity, recent studies have been focused on quantifying diffusivity within the gray matter itself (Walhovd et al., 2014; Assaf, 2019). The most common measure of local tissue diffusivity within gray matter is mean diffusivity (MD), a measure of the average diffusion properties within each voxel’s underlying tissue (Basser et al., 1994; Pierpaoli et al., 1996).

Gray matter MD is associated with alterations in synaptic, glial, and dendritic density and activity, such as swelling, arborization, and synaptic pruning (Blumenfeld-Katzir et al., 2011; Le Bihan, 2012; Sagi et al., 2012; Crombe et al., 2018; Stolp et al., 2018). Additionally, previous work suggests that cortical and subcortical gray matter diffusivity are stronger predictors of cognitive performance than volumetric measures across the lifespan (Kantarci et al., 2005; Jeon et al., 2012; Hong et al., 2013; Pereira et al., 2014; Weston et al., 2015; Callow et al., 2020). In the context of development, gray matter MD is generally negatively associated with age and better cognitive performance and is thought to represent increased myelination and axonal and neural density (Mah et al., 2017; Fjell et al., 2019; Callow et al., 2020). Meanwhile, in older adults, age and disease progression are usually positively associated with gray matter MD, which is believed to result from a general decline in dendritic and synaptic density (Ray et al., 2006; Pereira et al., 2014; Weston et al., 2015; Salminen et al., 2016; O’Shea et al., 2016; Langnes et al., 2019).

A recent report suggests that 50% of dementia cases could be prevented or delayed by reducing or eliminating risk factors through lifestyle behaviors, such as increased physical activity (Barnes and Yaffe, 2011; Kuehn, 2020). Despite a lack of pharmacological solutions for dementia, a growing body of longitudinal research suggests that individuals with MCI who are more physically active are at a reduced risk of cognitive decline and dementia progression (Blondell et al., 2014). Additionally, there is evidence to suggest that exercise training provides global cognitive benefits for older adults with MCI (Song et al., 2018). Meanwhile, several neuroimaging studies suggest that in conjunction with cognitive improvements, aerobic exercise training and higher cardiorespiratory fitness in individuals with MCI are associated with improvements in neural efficiency (Smith et al., 2013), enhanced functional connectivity (Chirles et al., 2017; Won et al., 2021), preservation of cortical thickness (Reiter et al., 2015), reductions in resting cerebral perfusion (Alfini et al., 2019), and protection of white matter tract integrity (Tarumi et al., 2020).

Few exercise intervention neuroimaging studies have been conducted in people with MCI, and no studies to date have been characterized the effects of exercise training on gray matter microstructure. Only two studies have been evaluated how cardiorespiratory fitness and exercise training are related to gray matter microstructure in healthy older adults. These studies are focused found hippocampal MD was negatively associated with cardiorespiratory fitness in those 80 years or older (Tian et al., 2014) and that 6-months of aerobic exercise training led to a reduction in hippocampal gray matter MD (Kleemeyer et al., 2016). However, both studies were limited to testing cognitively healthy older adults, and neither measured MD changes in cortical gray matter regions other than the hippocampus. Therefore, the goal of the current study is to determine how an exercise training intervention may differentially affect cortical and subcortical gray matter diffusivity in individuals diagnosed with MCI relative to cognitively healthy controls (HC). Previous work suggested that individuals with MCI had higher cortical diffusivity and experience differential benefits from exercise training than healthy controls (Smith et al., 2013; Chirles et al., 2017; Alfini et al., 2019). Given this, we hypothesized that individuals with MCI would exhibit higher gray matter diffusivity at baseline and a greater change in gray matter MD following the exercise intervention. To test these hypotheses, we assessed the effects of a 12-week supervised walking intervention on whole-brain gray matter MD in older adults classified as cognitively healthy vs. those diagnosed with MCI.

## Materials and Methods

### Subjects

This study was completed in accordance with the Helsinki Declaration and was approved by the Institutional Review Board of the Medical College of Wisconsin. Community-dwelling older adults between the ages of 60 years and 88 years were recruited from the surrounding area through study fliers, physician referrals, and in-person informational sessions at retirement communities and recreation centers. Interested participants underwent telephone screening to determine eligibility. All qualified participants provided written informed consent, received physician approval to participate in an exercise intervention, and underwent neurological assessment to confirm eligibility.

### Eligibility and Exclusion Criteria

A complete list of exclusionary criteria and prohibited medications can be found in our previous study (Smith et al., 2013). In short, participants were excluded if they engaged in moderate intensity physical activity more than 3 days per week within the past 6 months, had a history of neurological illnesses or untreated DSM-IV Axis I psychiatric illness (including major depression), had a medical illness that could potentially influence brain function, impaired activities of daily living, or any MRI contraindications.

### Neuropsychological Testing

A comprehensive neuropsychological test battery was conducted before and after the exercise intervention, followed by an exercise stress test and MRI scan on a different day. The neuropsychological test battery evaluated several aspects of cognition, and a full report can be found in our previous study (Smith et al., 2013). The battery included the Geriatric Depression Scale, Mattis Dementia Rating Scale 2 (DRS-2), Rey Auditory Verbal Learning Test (RAVLT), phonemic Controlled Oral Word Association Test (COWAT), semantic animal fluency test, and the Clock Drawing Test. Alternate test forms were used for each time point when possible, including for the RAVLT and DRS-2. MCI diagnosis was based on the criteria set forth by NIH-Alzheimer’s Association workgroup on the diagnosis of MCI due to Alzheimer’s (AD; Albert et al., 2011), and was defined by: (1) subjective concerns regarding change in cognition; (2) impairment in one or more cognitive domains; (3) preservation of independence in activities of daily living; and (4) not demented.

### Cardiorespiratory Fitness Testing

Extensive details on the cardiorespiratory fitness testing procedure can be found in our previously published study (Smith et al., 2013). In short, prior to and following the exercise intervention, participants completed a submaximal exercise test on a motorized treadmill (General Electric, Milwaukee, WI, USA) using a modified Balke-Ware protocol following the American College of Sports Medicine guidelines. Exercise testing was terminated at 85% of age predicted maximal heart rate (220-age) and VO_2peak_was estimated from the highest relative VO_2_ obtained (ACSM, 2006).

### Exercise Intervention

After baseline testing, all participants completed a 12-week walking exercise intervention that included four 30-min sessions of moderate-intensity treadmill walking per week. These sessions were performed at local recreation centers and consisted of small groups that were supervised by certified exercise trainers. All exercise training sessions began and ended with a 10-min light walking warm-up and cool-down. The exercise session intensity increased progressively to a heart rate reserve of 50–60% by the fifth week, at which point intensity was maintained for the rest of the intervention. During the exercise sessions, heart rate (Polar monitor) and ratings of perceived exertion (RPE; 6–20 scale; Borg, 1982) were measured to track training intensity and customize progressions of treadmill speed and grade for each participant to promote aerobic fitness improvements.

### MRI Acquisition

All MRI data was acquired using a 3.0 Tesla GE (Waukesha, WI) MR scanner. A high resolution T1-weighted anatomical brain image was acquired using a 3D Spoiled Gradient Recalled at steady state using the following sequence parameters, matrix = 256 × 224, field-of-view (FOV) = 240 mm, pixel size = 1 × 1 mm2, slices = 144, slice thickness = 1.0 mm, repetition time (TR) = 9.6 ms, echo time (TE) = 3.9 ms, inversion time (TI) = 450 ms, flip angle = 12°. Diffusion images were acquired using a Dual Spin Echo with 19 non-collinear diffusion-weighted acquisitions with *b* = 900 s/mm^2^ and a single T2-weighted *b* = 0 s/mm^2^ acquisition (b0 image) (FOV = 240 mm, voxel size = 0.9375 × 0.9375 × 3 mm^3^; TR/TE = 11,000/84 ms, matrix = 128 × 128, flip angle = 90°, and a bandwidth of 1,221 Hz/Px comprising 96 3-mm-thick slices).

### Anatomical Image Preprocessing

Anatomic image processing was performed with the FreeSurfer image analysis suite^1^ (version 6.0). Initially, the cross-sectional “recon-all” processing stream was implemented to perform initial intensity normalization, motion correction, and computation of the transformation to standard space, followed by non-brain tissue removal, cortical reconstruction, and volumetric segmentation of cortical and subcortical structures. Freesurfer’s longitudinal stream was then employed to reduce variability and improve skull stripping and segmentation performance across time points (Reuter et al., 2012). All reconstructed data were visually checked for skull removal and segmentation accuracy. No manual intervention with the MRI data was needed.

### Diffusion-Weighted Image Preprocessing

Diffusion-weighted images were processed using MRtrix3 commands or MRtrix3 scripts (Tournier et al., 2019) that link the FMRIB Software Library (FSL v6.0.1; Image Analysis Group, FMRIB, Oxford, UK^2^; Smith et al., 2004). First, physiological noise due to the thermal motion of water molecules was removed (Veraart et al., 2016), followed by the removal of Gibbs ringing artifacts (Kellner et al., 2016), and then brain extraction using the dwi2 mask command. A recently developed distortion correction and intensity-based registration method (Synb0) was used to correct b0 inhomogeneities (Schilling et al., 2020). This method uses a deep learning synthesis approach where an undistorted b0 image is synthesized from a distorted b0 and a T1 image to provide FSL’s Topup command with the information necessary to correct the distorted diffusion data. This method performs robust distortion correction similar to the state-of-the-art techniques that require blip-up blip-down acquisitions (Schilling et al., 2020). With the results from Topup, eddy current correction was then performed (Andersson and Sotiropoulos, 2016), followed by bias field correction (Tustison et al., 2014). Finally, the dwi2 tensor command was used to fit a diffusion tensor model to each brain voxel, as well as FA and MD values.

### Gray Matter Voxel-Wise Analysis

Gray matter voxel-wise analysis was performed in MNI space. First, MD images were nonlinearly transformed into MNI space in a two-step process using Advanced Normalization Tools (ANTS; Avants et al., 2008). For each registration, a linear rigid registration was applied first, followed by a diffeomorphic transformation using the Symmetric Normalization (SyN). The first step consisted of registering the b0 image to its respective T1 scan and then registering the T1 scan to MNI space. These two estimated registration maps were combined, and MD images were transformed into MNI space. Transformed MD images were concatenated into a single 4D image, and spatial smoothing with a 6-mm FWHM Gaussian kernel was applied.

To restrict the analysis to gray matter (GM) voxels and reduce the likelihood of partial volume effects, global GM and cerebrospinal fluid (CSF) masks were created. The GM mask was constructed by processing each T1 image with FSL’s FAST segmentation tool (Smith, 2002), which obtained binary segmentation images of GM, CSF, and white matter (WM). The GM image was warped into MNI space using the previously calculated T1 to MNI space registration maps. With each GM image in MNI space, a global GM mask was then created by restricting the mask to voxels in which at least 90% of subjects’ GM masks were included. As discussed in Henf et al. (2018), when examining GM MD in older adults or individuals with neurodegenerative disease, it is essential to consider partial volume effects that might arise from CSF contamination (Henf et al., 2018). To control for CSF contamination in the GM mask, a free water CSF-like mask was created using MRtrix3 Tissue^3^, a fork of MRtrix3 (Tournier et al., 2019). MRtrix3 Tissue is a method that allows 3-tissue constrained spherical deconvolution results from single-shell diffusion data. The three tissue compartments determine the contribution of free water CSF-like, WM-like, and GM-like signal within each voxel and has been shown to exhibit high reliability, particularly for estimating the contribution of free water CSF-like diffusion (intraclass correlation above 0.95; Newman et al., 2020). The three tissue compartment response functions were created and estimated for each diffusion scan, and a study wide response function for each tissue type was created. Three tissue compartment images were computed for each diffusion scan using the study wide response function. These three tissue compartment images were then normalized to sum to 1 on a voxel-wise basis to provide a three-tissue signal fraction map (providing the percent of GM-like, CSF-like, and WM-like signal in each voxel). As previously suggested (Newman et al., 2020), each subject’s CSF mask was then thresholded to only include voxels considered to have 50% or more CSF-like signal. All subjects’ CSF masks were then warped into MNI space using the same warp used on the MD images. Once the CSF images were in MNI space, a global CSF mask was created by including all voxels in which 10% or more of the subjects had identified a voxel as CSF in their individual mask. The final global GM mask was established by removing any voxels that overlapped with the global CSF mask and was then used for the following voxel-wise analysis.

### Statistical Analysis

For all analyses, significance was determined using a two-tailed alpha < 0.05. First, all between-group differences in demographic characteristics were compared using independent sample *t*-tests for continuous variables and chi-squared tests for categorical variables. A repeated-measures analysis of variance was then used to test for exercise-induced aerobic fitness and neurophysiological performance changes. A voxel-wise analysis using the global GM mask, and age as a covariate, was performed using AFNI’s linear mixed-effects modeling program 3dLME to determine within and between-group differences over time. The 3dLME program was used due to its flexibility and ability to compute repeated measures analysis. Using effective smoothness (ACF estimates) and first-order nearest neighbor clustering, we controlled for multiple comparisons and reduced the risk of Type-I errors (Cox et al., 2017). A family-wise error (FWE) corrected significance threshold was set at *p* < 0.05 (voxel-level *p* < 0.05, cluster-level α = 0.05), which maintained clusters ≥936 contiguous voxels. All significant clusters were anatomically identified with FSL’s atlasquery function using the MNI Structural Atlas, which gives the probability of a voxel or cluster being a member of a labeled region within an atlas. As a follow-up analysis, partial correlation analysis was employed in JASP [JASP Team (2020), Version 0.13.1^4^] to determine the association between changes in MD scores from pre- and post-exercise training and changes in cognitive performance, controlling for age. Cognitive performance scores found to have a significant main effect of Time or Group × Time interaction effect were included in this analysis.

## Results

### Participants

Detailed information about study recruitment can be found in our previously published work (Smith et al., 2013). In short, 407 individuals responded to study advertisements, of which 39 started the exercise program and 35 older adults (17 MCI and 18 HC; aged 61–88) completed the walking intervention. Three participants were excluded from further analysis due to missing a diffusion imaging scan at either of the two testing time points (15 MCI and 17 HC). At baseline, those diagnosed with MCI and HC were not significantly different in age, sex, education, APOE genotype status, functional abilities, or cardiorespiratory fitness. However, despite overall low depression scores (within normal limits), individuals with MCI had higher depression scores (*t*_(26)_ = 2.9, *p* = 0.007; Table 1), which is commonly reported in MCI (Shahnawaz et al., 2013). Adherence to the exercise protocol was high ( 96%), and throughout the intervention, HR and RPE did not significantly differ between groups (see Smith et al., 2013).

**Table 1:** Baseline demographic information.

		Total sample	MCI	HC	Group differences
		(*n* = 32)	(*n* = 15)	(*n* = 17)	*p*-value
		Mean (SD)	Mean (SD)	Mean (SD)	
Demographics
	Age (years)	78.4 (6.8)	80.5 (5.6)	76.5 (7.0)	0.10
	Female (*n*, %)				0.16
	Education (years)	16.0 (2.6)	15.6 (3.1)	16.5 (1.9)	0.25
	APOE-*ε*4 Carriers	12	5	5	0.81
Cardiorespiratory fitness
	Baseline VO_2peak_ (ml/kg/min)	19.9 (3.9)	19.5 (5.2)	19.1 (6.6)	0.33
Depression
	Baseline GDS	4.8 (3.3)	5.3 (4.5)	3.2 (2.0)	**0.007**
Cognition	Baseline DRS-2	134.1 (11.2)	128.8 (13.3)	140.5 (2.5)	**0.002**
Activities of Daily Living
	Baseline Lawton IADL	4.7 (0.5)	4.7 (0.5)	4.7 (0.5)	0.87

*Notes: MCI, Mild Cognitive Impairment; HC, Healthy Control; APOE-ɛ4, apolipoprotein E epsilon 4 allele; VO_2peak_, peak rate of oxygen consumption in millimeters per kilogram per minute (ml/kg/min); GDS, Geriatric Depression Scale; DRS-2, Mattis Dementia Rating Scale-2; IADL, Instrumental Activities of Daily Living. Bold indicates *p* < 0.05*.

### Exercise Intervention Efficacy and Neuropsychological Performance

At baseline, the MCI group had worse performance than the HC group on all neuropsychological tests other than RAVLT Trial 1. After the exercise intervention, both groups had a significant improvement in VO_2peak_, *F*_(1,26)_ = 6.03, *p* = 0.021, RAVLT Trial 1, *F*_(1,30)_ = 16.83, *p* = 0.007 and the COWAT, *F*_(1,30)_ = 6.23, *p* = 0.018. A significant Group × Time interaction was also found for COWAT, *F*_(1,30)_ = 5.99, *p* = 0.020, with MCI exhibiting greater COWAT performance improvements than HC. There were no additional main effects of Time or Group × Time interactions for any other neuropsychological tests (see Table 2).

**Table 2:** Cardiorespiratory fitness and neuropsychological performance data.

	Total sample (*n* = 32)		MCI (*n* = 15)		HC (*n* = 17)		Time	Group × Time
	Before	After	Before	After	Before	After
	Mean (SD)	Mean (SD)	Mean (SD)	Mean (SD)	Mean (SD)	Mean (SD)	*p*-value ($\eta_{p}^{2}$)	*p*-value ($\eta_{p}^{2}$)
**Cardiorespiratory fitness**
VO_2 peak_ (ml/kg/min)	19.1 (5.8)	21.0 (3.8)	18.7 (3.8)	21.0 (3.2)	19.4 (7.3)	21.1 (4.4)	**0.021 (0.19)**	0.730 (0.01)
**Cognitive**
RAVLT-Trial 1	4.7 (2.1)	5.7 (1.8)	4.4 (1.8)	5.9 (1.6)	4.9 (2.4)	5.5 (2.0)	**0.007 (0.22)**	0.228 (0.05)
RAVLT-Trial 1-5	42.8 (13.8)	45.3 (13.8)	38.7 (11.6)	42.9 (11.8)	46.4 (15.0)	47.5 (15.4)	0.122 (0.10)	0.287 (0.04)
RAVLT IR	8.8 (4.1)	8.4 (4.3)	6.8 (3.7)	7.5 (4.3)	9.9 (4.5)	10.1 (3.6)	0.272 (0.04)	0.520 (0.01)
RAVLT DR	8.4 (4.5)	8.3 (4.6)	6.8 (4.1)	9.5 (4.6)	6.9 (4.4)	9.7 (4.5)	0.818 (0.01)	0.917 (0.01)
Clock drawing	1.6 (1.1)	1.4 (0.8)	2.3 (1.1)	2.0 (0.8)	1.6 (1.1)	1.4 (0.8)	0.093 (0.09)	0.914 (0.01)
COWAT	36.3 (12.0)	39.1 (13.7)	34.1 (11.2)	40.0 (14.5)	38.3 (12.8)	38.4 (13.4)	**0.018 (0.17)**	0.020 (0.17)
Animal fluency	16.9 (6.7)	17.0 (8.0)	14.8 (6.5)	14.1 (8.3)	18.8 (6.5)	19.5 (7.1)	0.987 (0.01)	0.411 (0.02)

*MCI, Mild Cognitive Impairment; HC, Healthy Control; RAVLT, Rey Auditory Verbal Learning Test; Trial 1, Trial 1–5; IR, Immediate Recall; DR, Delayed Recall; COWAT, Controlled Oral Word Association Test; VO_2 peak_, peak rate of oxygen consumption; *p*-values and effect size ($\eta_{p}^{2}$) reflect the Time and Group × Time effects from repeated measures ANOVA; Bold indicates *p* < 0.05*.

### Voxel-Wise Analysis of Group × Time Interaction on Gray Matter Diffusivity

**Figure 1:**
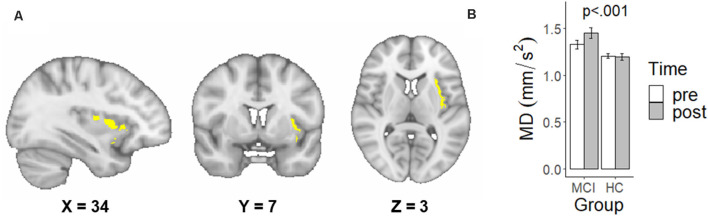
A significant family-wise error corrected interactive effect of Group × Time on mean diffusivity (MD) in the left insular cortex. **(A)** Sagittal, coronal, and axial view of the significant interaction cluster and the location of peak difference in MNI space using radiological convention. **(B)** Mean and standard deviations of raw MD values extracted from the significant interactioncluster, not controlling for age.

The Group × Time voxel-wise analysis resulted in a single significant cluster identified as predominantly the left insular cortex (42.2%; Figure 1A). Adjusting for age, a significant Group × Time interaction, *F*_(1,28)_ = 29.00, *p* < 0.001 was found for MD in this left insular cluster. Specifically, individuals with MCI had a significantly greater increase in left insular MD following the exercise intervention compared to HC (Figure 1B).

### Voxel-Wise Analysis of Main Effects of Time and Group on Gray Matter Diffusivity

**Figure 2:**
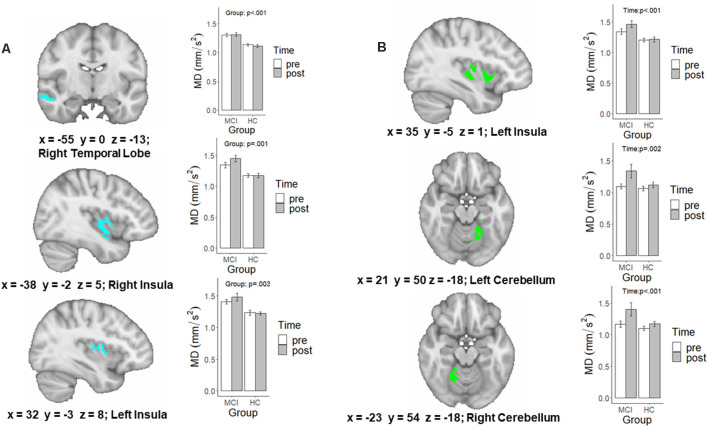
A map of the significant family-wise error (FWE) and age-corrected voxel-wise analysis of the **(A)** main effect of Group and **(B)** main effect of Time. Extracted values in the bar graph are raw diffusion values and not adjusted for age. p-values are the result of running these raw diffusion values through a similar linear mixed effects model, in which age was controlled for.

A detailed account of the age-adjusted main effect of Group and Time clusters can be found in Table 3. The initial main effect of Time voxel-wise analysis, resulted in three significant clusters located in the left (*F*_(1,28)_ = 10.98, *p* = 0.002) and right anterior and adjacent cerebellar lobule (*F*_(1,28)_ = 11.88, *p* = 0.002) and left insular cortex (*F*_(1,28)_ = 22.35, *p* < 0.001; see Figure 2). In each cluster, MD was greater following the intervention. Meanwhile, the main effect of Group voxel-wise analysis resulted in three significant clusters located in the right (*F*_(1,28)_ = 12.48, *p* = 0.001) and left insular (*F*_(1,28)_ = 10.74, *p* = 0.003) and the right temporal lobe (*F*_(1,28)_ = 22.6, *p* < 0.001), see Figure 2. In all three clusters, those with MCI had higher MD than HC.

**Table 3:** Significant mean diffusivity values for the Group, Time, and Group × Time voxel-wise analysis clusters.

Cluster	Cluster region	Peak location			Volume	MCI (*n* = 15)		HC (*n* = 17)	
		*x*	*y*	*z*	(voxels)	Before	After	Before	After
	**Group × Time Interaction**								
1	Left Insula	35.0	5.0	2.0	1,588	1.33 (0.18)	1.45 (0.21)	1.21 (0.11)	1.20 (0.14)
									
	**Time Main Effect**								
2	Left Cerebellum	21.0	50.0	−18.0	2,184	1.10 (0.17)	1.34 (0.43)	1.06 (0.15)	1.12 (0.18)
3	Left Insula	35.0	−5.0	1.0	2,127	1.34 (0.18)	1.46 (0.21)	1.20 (0.13)	1.21 (0.15)
4	Right Cerebellum	−23.0	54.0	−18.0	1,199	1.68 (0.19)	1.40 (0.41)	1.10 (0.15)	1.17 (0.16)
									
	**Group Main Effect**								
5	Right Temporal Lobe	−55.0	0.0	−13.0	1,976	1.30 (0.12)	1.31 (0.14)	1.14 (0.09)	1.12 (0.10)
6	Right Insula	−38.0	−2.0	5.0	1,525	1.37 (0.14)	1.44 (0.25)	1.21 (0.15)	1.19 (0.10)
7	Left Insula	32.0	−3.0	8.0	1,159	1.27 (0.18)	1.36 (0.19)	1.12 (0.12)	1.11 (0.16)

*Notes. Mean and standard deviations of *r*aw mean diffusivity values extracted from significant family-wise error corrected Group, Time, and Group × Time clusters. Clusters produced using AFNI’s 3dLME procedure for a voxel-wise analysis, after adjusting for age. Regions are defined by the highest probability using the MNI Structural Atlas*.

### Association Between Changes in MD and Cognitive Performance

**Figure 3:**
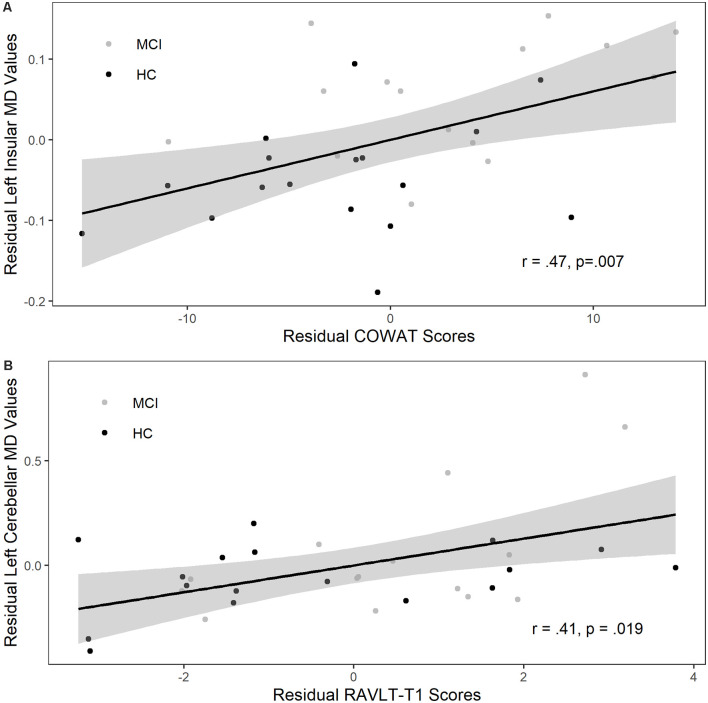
Change in gray matter (GM) diffusivity was positively associated with improved verbal fluency and episodic memory performance. **(A)** Residualized change in verbal fluency (COWAT, Controlled Oral Word Association Test) scores were significantly related to residualized change in left insular MD values, controlling for age. **(B)** Residualized change in rey auditory verbal learning test (RAVLT) Trial 1 scores were significantly related to residualized change in left cerebellar MD values, controlling for age. The two groups are healthy controls (HC; black symbols) and those diagnosed with Mild Cognitive Impairment (MCI; gray symbols). Gray shaded region represents 95% confidence interval.

Changes in MD values were extracted from the left insular interaction cluster and the left insular and right and left cerebellum main effect clusters, as defined by the age adjusted voxel-wise analysis, for both MCI and HC. Partial correlations suggested a significant positive relationship between increases in MD within the left insular interaction cluster and improvements in COWAT performance (*r* = 0.46, *p* = 0.007), but not changes in RAVLT-T1 performance (*r* = 0.22, *p* = 0.218), see Figure 3. Similarly, the increase in MD from the main effect of time left insular cluster was similarly positively associated with COWAT performance (*r* = 0.41, *p* = 0.02), but not RAVLT-T1 performance (*r* = 0.30, *p* = 0.09). Furthermore, training induced increases in MD values in both the left (*r* = 0.41, *p* = 0.019; see Figure 3) and right cerebellum (*r* = 0.36, *p* = 0.046) were associated with improvements in RAVLT-T1 performance, but not COWAT performance (*r* = 0.21, *p* = 0.243; *r* = 0.27, *p* = 0.14).

## Discussion

We found that a 12-week walking intervention significantly improved aerobic capacity (cardiorespiratory fitness), phonemic verbal fluency (measured by the COWAT), and immediate verbal recall performance (measured by the RAVLT-T1). We also found that a 12-week walking intervention led to a significant interaction effect with gray matter MD values of the left insular, finding individuals diagnosed with MCI exhibited greater increases in MD than HC. Additionally, we found exercise training was associated with increases in MD in the left insular cortex and right and left cerebellum for both MCI and HC. Further analysis revealed that these exercises training-related increase in left insular MD were significantly associated with verbal fluency improvements. In contrast, the increases in anterior and adjacent cerebellar MD were related to gains in RAVLT-T1 performance. Finally, we found MCI had greater cortical MD in the right temporal lobe and right and left insular compared to HC.

### Group Differences in Cortical Gray Matter Diffusivity

In the current study, individuals with MCI exhibited greater baseline MD values than HC in the left and right insular and right medial temporal lobe. This is consistent with previous work, showing individuals with MCI and AD generally exhibit higher cortical and subcortical gray matter MD at baseline than healthy controls in regions such as the hippocampus, entorhinal cortex, parietal cortex, precuneus, insula, frontal cortex, and temporal cortex (Müller et al., 2005; Ray et al., 2006; Rose et al., 2008; Scola et al., 2010; Weston et al., 2015, 2020; Lee et al., 2020; Torso et al., 2020). However, the differences in cortical gray matter MD appear to be less pronounced in less severely impaired individuals (Scola et al., 2010; Lee et al., 2020; Weston et al., 2020). Furthermore, some previous studies failed to control for partial volume effects due to CSF contamination, which is essential to control for in populations where there is potential for significant neurodegeneration and volume loss (Henf et al., 2018). We report higher cortical MD in the MCI group after controlling for CSF, free water contamination, and partial volume effects. These results are consistent with the general literature however, the more limited extent of our reported effects may be due to more strict processing steps and may also be due to most of our MCI participants being recently diagnosed and therefore, likely earlier in the disease course trajectory.

Many studies reporting elevated cortical MD, find these effects occur independently of the cortical thickness or volumetric changes in gray matter (Weston et al., 2015, 2020; Lee et al., 2020). While both gray matter microstructure and macrostructure are associated with age, they are generally unrelated when controlling for age, suggesting the two measures are sensitive to different underlying neurophysiological changes that may occur at different stages of aging and dementia progression (Zhao et al., 2019). For example, higher gray matter MD is believed to represent a breakdown in the microstructural barriers to diffusion, which is predicted to precede volumetric changes (Ly et al., 2014; Weston et al., 2015). Specifically, this reduction in microstructural barriers is believed to result from a loss of synapses and neurons, shrinkage of larger neurons, and increases in glial activity and neuroinflammation (Weston et al., 2015; Stolp et al., 2018; Lafrenaye and Simard, 2019; Zhao et al., 2019). Our findings of higher cortical gray matter MD and poorer cognitive performance in individuals with MCI are consistent with the evidence that MCI is a transitory state that involves distinct neurophysiological differences in brain function and structure compared to healthy older adults (Langa and Levine, 2014; Jongsiriyanyong and Limpawattana, 2018). Nevertheless, the timing and direction of changes in MD, and the mechanisms that determine these changes, may or may not always reflect a pathological process, and are not yet completely understood (Fortea et al., 2010; Ryan et al., 2013; Weston et al., 2015).

### Exercise Training Induced Changes in Cortical Gray Matter Diffusivity

Notably, following the exercise intervention, the participants diagnosed with MCI exhibited *increased* MD within the left insular cortex, which was associated with verbal fluency* improvements*. While higher gray matter MD generally associated with cognitive decline and thought to indicate disease progression, a recent large cross-sectional study in younger adults found that higher cortical MD in several regions, including the left insular, was associated with better empathizing and cooperativeness (Takeuchi et al., 2019). Additionally, several studies focusing on familial AD found that just prior to symptom onset, MD was reduced in various cortical and subcortical gray matter regions, such as the precuneus, insula, parietotemporal area, thalamus, putamen, and caudate (Fortea et al., 2010; Ryan et al., 2013). It was hypothesized that in the early presymptomatic stages of AD, water molecules normally diffuse through cellular barriers, as seen in healthy individuals. However, leading up to disease progression and symptom onset, diffusion becomes restricted (*lower MD*) due to cellular hypertrophy and inflammation in response to amyloid deposition. Finally, during the symptomatic phase, progressive cellular atrophy results in the breakdown of cellular barriers and a subsequent large increase in MD (Weston et al., 2015). Although our finding of exercise training-induced increases in left insular MD in the MCI individuals could indicate negative neurophysiological changes, these changes were associated with improvements in verbal fluency and marginally associated with verbal memory recall improvements. Therefore, it is more likely that exercise training-induced increases in MD within the insula could indicate improvements in underlying cellular integrity and reduced inflammation and cellular swelling. For example, in our recent article (Alfini et al., 2019), we found in this cohort that exercise training reduced left insular cerebral blood flow in individuals with MCI, but not healthy controls and that this change was also associated with improvements in verbal fluency. Reductions in hyperperfusion from exercise are hypothesized to result from the normalization of blood flow and oxygen availability due to cerebrovascular growth (Pereira et al., 2007; Alfini et al., 2019), leading to lower inflammation and improve cellular integrity (Wierenga et al., 2014). However, we controlled for the fast free water compartment in our analysis, which absorbs a large portion of perfusion effects and thus, helps limit contamination in the diffusion signal from CSF and the intravoxel incoherent motion of blood due to differences in capillary perfusion (Rydhög et al., 2017; Newman et al., 2020). As an additional check, we also found no association between the subsample of perfusion values and MD values or between change in perfusion and change in MD values extracted from the left insular interaction cluster. Therefore, while it is unlikely that increases in left insular MD result from alterations in perfusion specifically, it is possible both measures are sensitive to similar or synergistic underlying compensatory neurophysiological mechanisms that elicit the reported improved verbal fluency.

Additionally, we found that both HC and MCI exhibited increased MD within a slightly overlapping section of the left insular cortex and the left and right anterior and adjacent cerebellar lobule. Interestingly, the left insular cortex changes showed a consistent association with verbal fluency performance. In contrast, changes in the left and right anterior and adjacent cerebellar MD were associated with immediate verbal recall performance. Previous work has found that anterior cerebellar volume is positively associated with immediate and delayed verbal recall (Kansal et al., 2017) and is heavily involved in working memory in general (Desmond and Fiez, 1998; Ashida et al., 2019). Additionally, a meta-analysis found that the adjacent cerebellum is involved in verbal working memory and executive function (Stoodley and Schmahmann, 2009). Furthermore, recent work suggests that cerebellar deterioration and volume loss are associated with cognitive decline in individuals with MCI (Lin et al., 2020). In this same cohort, we have also recently shown that exercise training increased cerebellar connectivity in the HC (Won et al., 2021). Thus, these increases in cerebellar MD, which are associated with improvements in immediate verbal recall, may indicate some form of structural remodeling that could be consistent with improved neural efficiency and connectivity in the region. It is important to note that due to the nature of this study, it is not possible to determine if these effects are the result of protective or compensatory mechanisms.

The scaffolding theory of aging and cognition (STAC) suggests that compensatory brain processes are responsible for maintaining cognitive performance despite the accumulation of neural challenges (Park and Reuter-Lorenz, 2009). These same authors later revised the STAC theory (STAC-r) to account for factors, such as physical activity, that contribute to the rate of change in cognitive function (Reuter-Lorenz and Park, 2014; Cabeza et al., 2018). Previous work indicates that in healthy individuals and those with MCI, over-activation and altered connectivity patterns in various cortical and subcortical regions, including the insula, is associated with poorer cognitive performance (Yassa et al., 2011; Smith et al., 2013; Chand et al., 2017; Liu et al., 2018). Exercise training appears to reduce this hyperactivity and help regulate insular connectivity in individuals with MCI (Smith et al., 2013; Chirles et al., 2017). Therefore, it is possible that exercise training may help enhance neural efficiency through synaptic and dendritic pruning (Brockett et al., 2015). In fact, recent work suggests that in healthy aging glia remain dynamic and active in pruning and refining synaptic processes (Mostany et al., 2013; Hong et al., 2016), while it is not until the more advanced stages of disease progression that widespread microglial related loss of synapses occurs (Rajendran and Paolicelli, 2018). Given we found changes in diffusivity in both the MCI and HC group, it is possible that increases in cortical MD and cognitive performance following the exercise intervention could be due to increased glial related synaptic pruning to improve neural efficiency and connectivity. This increased level of glial activity and reduced synaptic and dendritic density might thus result in the higher MD and better cognitive performance observed (Le Bihan, 2012, 2014; Smith et al., 2013; Tsurugizawa et al., 2013; Hong et al., 2016; Chirles et al., 2017). However, given the lack of specificity of measures of cortical MD, additional animal and human studies are needed to determine the specific mechanisms that might have caused these changes.

### Potential Mechanisms

Interpreting the changes in gray matter MD remains challenging due to the isotropic nature of the underlying tissue. However, animal work suggests that wheel running can upregulate various neurotrophic factors such as brain-derived neurotrophic factor (BDNF), insulin-like growth factor (IGF-1), and vascular endothelial growth factors (VEGF), which in turn promote angiogenesis, synaptogenesis, and neurogenesis (Pereira et al., 2007; Voss et al., 2013; Duzel et al., 2016; Maass et al., 2016; Stillman et al., 2020). However, most of this animal work has focused on the effects of exercise training on the hippocampus. Yet, some animal work suggests that wheel running may have more widespread benefits associated with enhanced synaptic, dendritic, and astrocytic measures in various cortical brain regions associated with cognitive improvements, such as the hippocampus, prefrontal, perirhinal, and the orbitofrontal cortex (Brockett et al., 2015). Furthermore, a recent meta-analysis of randomized controlled exercise training studies in older adults suggests that exercise protects various cognitive domains that are not specific to the hippocampus and that these benefits were consistent for both healthy older adults and those with MCI (Northey et al., 2018). Nevertheless, while exercise training appears to protect brain structure and function in healthy individuals and those with MCI, there remains little evidence for how exercise training affects neurophysiology in individuals with MCI. In this same cohort, we have previously reported improvements in cognition and preservation of cortical thickness (Reiter et al., 2015), reduced cerebral blood flow (Alfini et al., 2019), and alterations in functional connectivity (Chirles et al., 2017) and neural efficiency (Smith et al., 2013). These findings suggest that exercise training elicits neurophysiological changes in both MCI and healthy older adults’ cortex and that exercise may afford these cognitive benefits through various, potentially synergistic mechanisms. Our finding of increased gray matter MD was associated with improved verbal fluency and immediate verbal recall performance. Thus, these changes could be related to structural remodeling, normalization of cerebrovasculature and inflammation, and pruning of unnecessary synaptic connections, which may lead to enhanced efficiency and the reported preservation of cognition. However, future studies will need to include a non-exercising control group and observe the effects of exercise training on gray matter MD and cognition over a greater period and conduct follow-ups to determine how these cortical microstructure changes may relate to underlying neurophysiology and disease progression.

### Strengths and Limitations

The following study makes several contributions to the current literature. Our research suggests that a supervised walking intervention can improve cardiorespiratory fitness, verbal fluency, and verbal memory in healthy individuals and those with MCI. Furthermore, we found increased left insular and cerebellar MD in both MCI and HC, with greater increases in left insular MD in the MCI group. These exercise-induced increases in cortical MD were also associated with improvements in verbal fluency and immediate verbal recall performance. While individuals diagnosed with MCI are at a critical stage of cognitive decline, less is known about how exercise training may impact neural network integrity in MCI compared to the well-document effects of exercise training in healthy older adults. Additionally, we used diffusion imaging of cortical gray matter, an imaging metric that has not previously been used in the exercise neuroscience literature, and that may be an earlier and more sensitive measure of underlying microstructural integrity than standard volumetric measures. Finally, we utilized a well-validated battery of neuropsychological assessments to determine associations between exercise training-related changes in gray matter diffusivity and changes in cognition.

Although this article makes several unique contributions to the existing literature, it does have limitations. The most obvious limitation is the lack of a non-exercising control group. Given the high fidelity of our exercise intervention (96% adherence and significant improvement in aerobic capacity), it is unlikely that the passage of time or non-specific intervention effects (e.g., social interaction) are responsible for these findings. Nevertheless, it is impossible to rule out these possibilities and thus, caution must be taken in the interpretation of these findings until they are replicated in a randomized controlled clinical trial. It is also important to note that while MD is sensitive to various underlying neurophysiological changes in gray matter tissue, it is not specific to any of them. Additionally, older single-shell diffusion imaging protocols and diffusion tensor models, such as the method we employed, are generally more susceptible to partial volume effects. However, we used the most current and advanced analysis pipelines to achieve strict tissue segmentation and free water elimination, which restricted the analysis to gray matter and reduce the impact of CSF partial volume effects. Nevertheless, diffusion imaging pulse sequences that incorporate multiple b-values should be included in future studies. These measures are more sensitive and specific to underlying neurophysiological changes in gray matter tissue. Our study sample was also primarily Caucasian and well-educated, and thus, caution is warranted when attempting to infer these findings to the broader population.

## Conclusion and Future Direction

In conclusion, a single-arm 12-week walking intervention significantly improved cardiorespiratory fitness, verbal fluency, and episodic memory performance in individuals with MCI and HC. Furthermore, left insular MD increased in both groups, but to a greater extent in the MCI participants, and the overall increase in left insular MD was associated with improvements in verbal fluency in both groups. The specificity of the associations between changes in cerebellar MD and improvements in Trial 1 learning on the RAVLT further suggest that the salubrious effects of exercise training may simultaneously impact multiple neural networks. These findings provide additional evidence that exercise training may help to preserve cognition in individuals with MCI, and new evidence for the possibility that these effects are associated with remodeling of the cortical gray matter microstructure. Future research is needed to determine the mediating effects of cortical gray matter MD on the relationship between exercise and cognitive performance in HC and those with MCI.

## Data Availability Statement

The raw data supporting the conclusions of this article will be made available by the authors, without undue reservation.

## Ethics Statement

The studies involving human participants were reviewed and approved by Institutional Review Board of the Medical College of Wisconsin. The patients/participants provided their written informed consent to participate in this study.

## Author Contributions

KN and JS conceived, planned, and supervised the experiment. DC analyzed the data with the help of JS. DC wrote the manuscript with support from JW, GP, LJ, NA-N, YK, KN, and JS. All authors contributed to the article and approved the submitted version.

## Conflict of Interest

The authors declare that the research was conducted in the absence of any commercial or financial relationships that could be construed as a potential conflict of interest.

## Footnotes

^1^http://surfer.nmr.mgh.harvard.edu/

^2^http://www.fmrib.ox.ac.uk/fsl/

^3^https://3tissue.github.io

^4^http://jasp-stats.org/
